# Antarctica eye study: a prospective study of the effects of overwintering on ocular parameters and visual function

**DOI:** 10.1186/s12886-018-0816-0

**Published:** 2018-06-25

**Authors:** Matthew H. Stahl, Alexander Kumar, Robert Lambert, Michael Stroud, David Macleod, Andrew Bastawrous, Tunde Peto, Matthew J. Burton

**Affiliations:** 1 University College London, London, UK; 2 King’s College London, London, UK; 3 University of Fribourg, Fribourg, Switzerland; 4 Department of Trauma & Orthopaedic Surgery, Royal Infirmary of Edinburgh, Edinburgh, Scotland; 5 University of Southampton Medical School, Southampton, UK; 6 Clinical Research Department, International Centre for Eye Health, London School of Hygiene & Tropical Medicine, London, UK; 7 Moorfields Eye Hospital NHS Foundation Trust, London, UK; 8 Queens University Belfast, Belfast, UK; 9 Department of Medicine, Wexham Park Hospital, Wexham, Slough, SL2 4HL UK

**Keywords:** Eye, Antarctica, White Mars, Altitude

## Abstract

**Background:**

In 2013 five polar explorers attempted to complete the first Trans-Antarctic Winter Traverse (TAWT). This study presents the ophthalmological findings for this group, who overwintered in Antarctica as part of the White Mars Human Science Protocol. Antarctic crews are exposed to extreme cold, chronic hypoxia and altered day-night cycles. Previous studies of Antarctic explorers have focused on the prolonged effect of ultraviolet radiation including the development of ultraviolet keratitis and accelerated cataract formation. This is the first study of its kind to investigate the effect of overwintering in Antarctica on the human eye.

**Methods:**

Pre and post-expedition clinical observations were made including visual acuity, contrast sensitivity, colour vision, auto-refraction, subjective refraction, retinal examination, retinal autofluoresence and retinal thickness, which were graded for comparison. During the expedition additional observations were made on a monthly basis including LogMAR visual acuity, autorefraction and intraocular pressure.

**Results:**

No significant differences between pre and post-expedition observations were found, including visual acuity, contrast sensitivity, colour vision, refraction, visual fields, intraocular pressure and retinal examination. There was a small but statistically significant decrease in retinal thickness across all regions of the retina, except for the macular and fovea, in all explorers. Intra-expedition observations remained within normal limits.

**Conclusion:**

Reassuringly, the human eye remains largely unchanged by exposure to the extreme conditions encountered during the Antarctic winter, however, further research is needed to investigate changes in retinal thickness. This may have implications for scientists who spend prolonged periods of time in the polar regions, as well as those who have prolonged exposure to the extreme cold or chronic hypoxia in other settings.

## Background

In 2013 five polar explorers attempted a Trans-Antarctic Winter Traverse (TAWT). Good visual function is critical for effective and safe activity in extreme environmental conditions such as those found on the Antarctic Plateau. Furthermore, any damage to visual function may have implications for future expeditions, to those who regularly overwinter in the polar regions, and to those who experience prolonged exposure to the extreme cold and chronic hypoxia.

To date, the majority of outdoor and expedition research has focused on mountain climbers reaching high altitude [1–3]. These are generally higher than the Antarctic Plateau, which was reached during the TAWT, however the duration at altitude tends to be much shorter for mountain climbers. Such studies of mountaineers operating in cold and high altitude environments have shown moderate reversible changes in visual function and ocular parameters due to hypoxia: intraocular pressure rises slightly [1], central corneal thickness can increase [2], retinal blood flow may change [3], and marked hyperopic shifts have also been noted, particularly in the presence of previous radial keratotomy [4]. Moreover, at very high altitudes, a characteristic haemorrhagic retinopathy can develop [5–7].

In the early twentieth century during the heroic age of Antarctic exploration, polar explorers reportedly commonly suffered from ultraviolet (UV) keratitis (snow blindness) and the medical supplies taken on polar expeditions included ophthalmic preparations accordingly [8, 9]. Scant further studies have reported effects due to the prolonged UV radiation exposure including the development of UV keratitis, maculodystrophy, corneal dystrophy and accelerated cataract formation [10, 11]. A single study involving 24 Ukrainian Antarctic expedition personnel between 2011 and 2013 found specific changes including parenchymatous-endothelial keratopathy, nuclear-posteriorcapsular phacopathy and maculopathy, with morphological signs of acute solar retinopathy present in more than 50% winterers [11]. The TAWT afforded a new opportunity to study the effects of the winter in more detail, offering a different sort of environmental challenge, where UV exposure during the winter months is minimal. More recently telemedicine has been used to help diagnose ophthalmic conditions however, there is limited ophthalmic diagnostic equipment available in the health and medical clinics in Antarctic bases [12]**.**

Since Antarctica and this expedition in particular are often used as space analogue research environments, it is worth noting that significant changes have also been reported to develop in the eyes of astronauts, particularly those experiencing long-duration spaceflight [9]. Subjective changes in near vision were particularly common. Optic nerve oedema was also reported, possibly due to venous congestion in the brain [13]. As these specific effects were probably the result of prolonged microgravity, in relation to ophthalmological research, the TAWT did not provide an analogue for spaceflight. We set out to investigate the effect of overwintering in Antarctica on the human eye, examining extreme cold combined with the high altitude experienced during the TAWT on visual function and ocular parameters.

## Methods

### The expedition

Five polar explorers attempted the first Trans-Antarctic Winter Traverse. The team set out on March 21st 2013, aiming to cross the entire Antarctic continent. Their attempt was halted on June the 18th after 335 km. They progressed no further into Antarctica and remained in the same location until late November 2013.

### Clinical assessments: pre and post-expedition

Prior to leaving the UK for Antarctica, members of the expedition underwent a detailed ocular assessment in December 2012. These observations were then repeated at the end of the expedition when the team returned to the UK in November 2013. Team members were asked about any prior ocular problems, eye surgery, including refractive surgery, contact lens or spectacle use and their general health.

The pre and post-expedition clinical assessment included the following components: uncorrected, best corrected (with glasses if available) and pinhole visual acuity was measured using a standard ETDRS LogMAR backlit chart. Contrast sensitivity was measured using a Pelli-Robson Chart. Colour vision was assessed using an Ishihara chart. Visual acuity was re-measured using a prototype of Peek Acuity, an Android-based smartphone visual acuity test [14, 15]. Visual fields were tested using the Humphrey 24–2, full threshold protocol. Autorefraction was performed using a Nidek 510A and the hand held “SureSight” (Welch Allyn), followed by a subjective refraction.

The anterior segment was examined using a slit-lamp. Intraocular pressure (IOP) was measured by Goldman tonometry and a TA01i iCare (iCare). Pupils were dilated with tropicamide 1% and the retina examined at the slit-lamp. The retina was examined at the slit-lamp using an indirect lens (90D for the retina, 66D for the macular). Retinal photographs of the disc, macular and vascular arcades were taken using the standard retinal camera (Topcon 50DX) and repeated using a PanOptic ophthalmoscope (WelchAllyn) mounted on a Samsung S3 smartphone. Retinal autofluorecsence was measured using Spectralis OCT (Heidelberg Engineering). Retinal photographs were graded in a masked manner at the Moorfields Reading Centre for changes in the optic disc appearance, vessel calibre and signs of retinopathy.

### Clinical assessments during the expedition

The expedition team doctor took serial measurements on a four-weekly basis during the course of the expedition. These included LogMAR visual acuity using Peek Acuity, IOP measured (iCare) and autorefraction (SureSight).

### Analysis

Statistical analysis was performed using SPSS version 24. Wilcoxon signed rank test was used for the comparison of pre and post-expedition measurements. A Spearman’s rank correlation coefficient was calculated between IOP, VA and refractive measures recorded during the expedition and time spent in Antarctica. Data was expressed graphically either as mean +/− 95% confidence interval or the mean overlaying individual data.

## Results

### The expedition

The team set out on March 21st 2013 aiming to complete the first ever TAWT. They officially halted their attempt on June 18th of the same year, after travelling 335 km into the Antarctic continent, when they encountered a large crevasse field. They progressed no deeper into the Antarctic continent and retreated to a new position at an altitude of 2752 m, in order to make a semi-permanent camp for the winter. This allowed them to conduct their experiments until their return to the Antarctic coastline and departure from Antarctica in late November 2013. There was significant day to day variation in the amount of time each explorer spent outdoors. The median time explorer A spent outside: 101 min (range 60–302.4), explorer B: 189 min (range 107–381), explorer C: 210 min (105–366), explorer D: 70 min (8–235), explorer E: 84 min (56–180). This time was mostly spent doing light work.

Outside conditions experienced during intra-expedition measurements beginning in June: average temperature − 37 °C; minimum temperature − 55 °C; average wind speed 27 knots; maximum wind speed 63 knots; average barometric pressure 704 mbar; minimum barometric pressure 663 mbar; permanent altitude 2750 m.

### Participants

All explorers were Caucasian males. Their mean age at the pre-expedition assessment was 37 years (SD 10.3), with an age range of 28–54 years. Explorer E was unable to attend the post-expedition assessment.

### Visual acuity

**Fig. 1:**
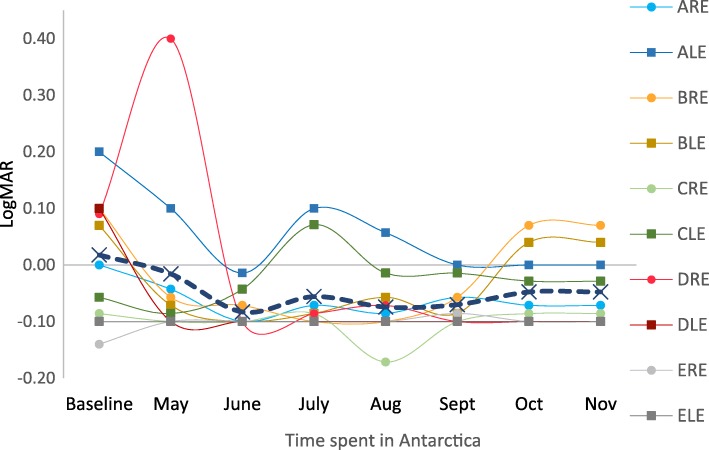
Individual and mean visual acuity measured during the expedition. ARE: explorer A right eye, ALE: explorer A left eye, etc.

The greatest change in a single eye between pre and post-test VA was a worsening of vision by 0.08 LogMAR units. There was no pre- and post-expedition change in 1 eye, an improvement in VA in 3 eyes and a worsening of VA in 4 eyes. There was not a statistically significant difference at the 5% level between pre- and post-expedition visual acuity (*P* = 0.61) (Table 1). The measurements taken during the expedition showed fluctuations in visual acuity in all participants between 0.10 and − 0.10 LogMAR units (Fig. 1). The first measurement for explorer D was 0.4 LogMAR units in one eye, which improved over the next month and remained stable throughout the expedition. This explorer had previously had refractive surgery (LASIK) in 2011, however, he did not recall any subjective change in visual acuity. This change did not coincide with any change in any other measured ocular parameter. There was no statistically significant association between visual acuity and time spent in Antarctica (*r* = − 0.25, *p* = 0.55).

**Table 1:** Pre- and post-expedition visual acuity, refraction and intra-ocular pressure

Explorer		LogMAR VA RE	LogMAR VA LE	Refraction RE			Refraction LE			IOP RE	IOP LE
				Sph	Cyl	Axis	Sph	Cyl	Axis		
A	Pre	− 0.14	− 0.18	0.25	−1.5	60	0.25	−2	92.5	10	10
	Post	− 0.14	− 0.1	0.25	−1	60	0	−1.75	90	9	10
	Diff	0	0.08	0	0.5	0	0.25	0.25	−2.5	−1	0
B	Pre	−0.14	−0.20	0	−0.25	100	−0.25	− 0.25	55	12	12
	Post	−0.16	−0.14	− 0.25	−0.25	135	−0.25	− 0.25	55	12	12
	Diff	−0.02	0.06	0.25	0	35	0	0	0	0	0
C	Pre	−0.18	−0.24	0.25	−0.25	85	−0.25	−1	70	15	14
	Post	−0.16	− 0.26	0.25	− 0.5	26	− 0.25	−0.5	67	17	17
	Diff	0.02	−0.02	0	−0.25	− 59	0	0.5	−3	2	3
D	Pre	−0.22	− 0.24	0.25	− 0.25	180	0.5	−0.25	140	20	20
	Post	−0.28	− 0.22	0.25	− 0.25	30	− 0.25	− 0.25	150	16	16
	Diff	−0.06	0.02	0	0	−150	−0.75	0	−10	−4	−4
E	Pre	−0.2	−0.24	0	−0.25	100	0.25	0	0	15	14
	Post	–	–	–	–	–	–	–	–	–	–
	Diff	–	–	–	–	–	–	–	–	–	–

*Pre* pre-expedition, *Post* post-expedition, *Diff* difference, *VA* visual acuity, *RE* right eye, *LE* left eye, *Sph* Sphere, *Cyl* Cylinder, *VA* visual acuity, *RE* right eye, *LE* left eye, *IOP* intra-ocular pressure

### Colour vision

There was no change in pre- and post-expedition colour vision in both eyes of explorers B, C and D with all three reading 21 / 21 of the Ishihara plates on both occasions. Colour vision was not measured in explorer A or E.

### Contrast sensitivity

There was some weak evidence of an improvement in pre- and post-expedition contrast sensitivity which was not statistically significant at the 5% level (*P* = 0.066). The largest change was an increase of 0.3 Log units. There was a slight improvement in the contrast sensitivity of 4 eyes and no change in 4 eyes.

### Refraction

**Fig. 2:**
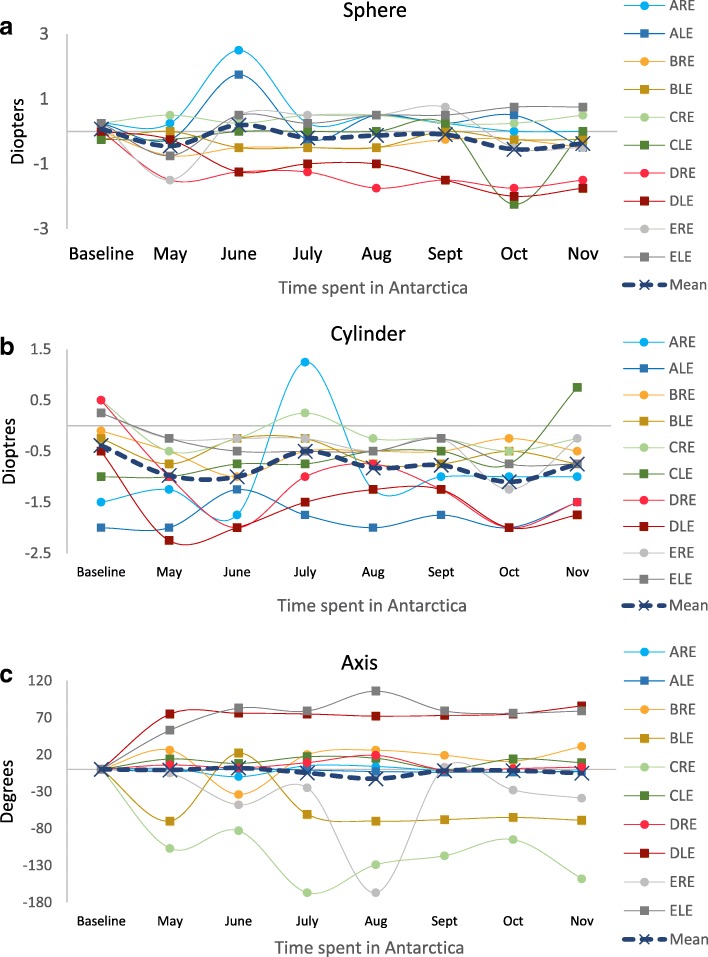
Monthly auto-refraction carried out during the expedition. ARE: explorer A right eye, ALE: explorer A left eye, etc. **a** sphere during expedition. **b** cylinder during expedition. **c** Change in axis. First measurement is baseline and subsequent data points represent change from baseline

There was no pre- and post-expedition change in 5 eyes in spherical refraction with a small positive shift in 1 eye and a small negative shift in 2. There was no change in 4 eyes in cylindrical refraction with a small positive shift in 3 eyes and a small negative shift in 2. There were some minor changes in pre- and post-expedition axis with no change in axis in 2 eyes, a positive shift in 1 and a negative shift in 5. There was one large pre- and post-expedition shift in axis of − 150 degrees in the right eye of explorer D. There was no similar intra-expedition change found. There was no statistically significant difference at the 5% level between pre- and post-expedition subjective refraction: sphere (*p* = 0.10), cylinder (*p* = 0.19), and axis (*p* = 0.61) (Table 1). During the expedition there was no significant association between cylinder (*r* = − 0.214, p = 0.61) or axis (*r* = − 0.667, *p* = 0.071) and time spent in Antarctica (Fig. 2). There was a small overall negative mean change in spherical refraction during the expedition. A negative association between sphere and time spent in Antarctica was found that was not statistically significant at the 5% level (*r* = − 0.619, *p* = 0.102).

### Visual fields

There was no statistically significant difference between mean pre- and post-expedition measurements of visual fields index (*p* = 0.80), mean deviation (*p* = 0.90), or pattern standard deviation (*p* = 0.40).

### Anterior segment examination

Clinical examination of the anterior segment revealed no pre- or post-expedition anterior segment pathology in any of the explorers**.**

### Intraocular pressure

**Fig. 3:**
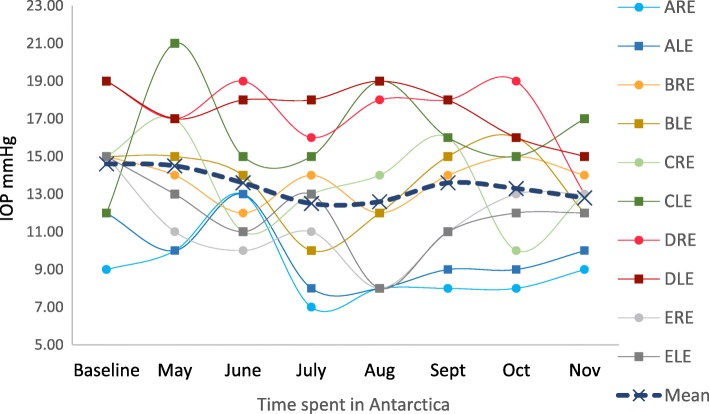
Individual monthly measurements of intra-ocular pressure taken during the expedition. ARE: explorer A right eye, ALE: explorer A left eye, etc.

The pre- and post-expedition IOP increased in 2 eyes, decreased in 3 eyes and did not change in three eyes. All changes were minor and within normal physiological limits. There was no statistically significant difference at the 5% level between pre- and post-expedition IOP (*P* = 0.498) (Table 1). Intra-expedition IOP remained within the normal range (≤21mmHg) for every explorer (Fig. 3). There was no statistically significant association found between IOP and time spent in Antarctica (*r* = − 0.50, *p* = 0.25).

### Retinal examination

**Fig. 4:**
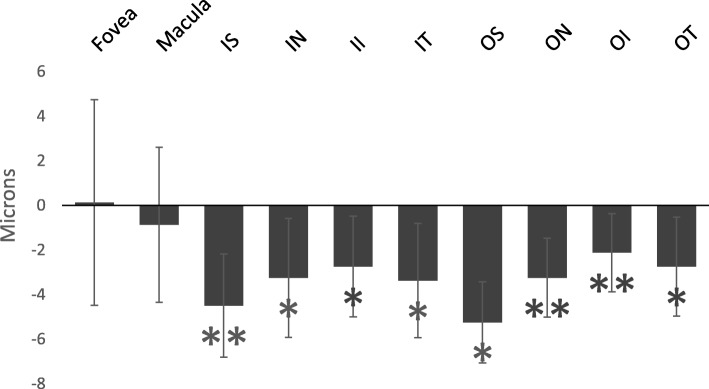
Pre- and post-test mean change in retinal thickness. Whiskers: 95% confidence interval. *: *P* < 0.05.**:*p* < 0.01. Percentages indicate pre- and post-expedition change. IS: inner-superior retina, IN: inner-nasal retina, II: inner-inferior retina, IT: inner-temporal retina, OS: outer-superior retina etc. Explorer E was not included due to missing data

No retinal pathology was identified either on clinical examination or on review of the photographs in any of the explorers. There was no statistically significant difference between the pre and post-expedition foveal retinal thickness (*p* = 0.83) or the macular retinal thickness (*p* = 0.55). All other areas of retina showed a small but statistically significant decrease in retinal thickness (range − 2.13 - -5.25 μm). There was a mean decrease of 4.50 μm in the inner-superior retina (*p* = 0.012), 3.25 μm in the inner-nasal retina (*p* = 0.042), 2.75 μm in the inner-inferior retina (*p* = 0.034), 3.38 μm in the inner-temporal retina (*p* = 0.027), 5.25 μm in the outer-superior retina (*p* = 0.011), 3.25 μm in the outer-nasal retina (*p* = 0.017), 2.13 μm in the outer-inferior retina (*p* = 0.027), and 2.75 μm in the outer-temporal retina (*p* = 0.035) (Fig. 4).

It was not possible to assess the retinal vessel calibre in explorer A due to a magnification error when taking the photo. There was no statistically significant difference at the 5% level between pre and post-expedition central retinal arteriolar equivalent (*p* = 0.69), central retinal vein equivalent (*p* = 0.600), or the aterio-venous ratio (*p* = 0.14) of the remaining three participants.

## Discussion

There are many potential causes of ocular damage in an extreme environment such as Antarctica. These include factors such as exposure of the cornea to the extreme cold, high altitude, excessive UV light, trauma and a diet lacking in nutrients beneficial to the eye [9, 11]. Although the data need to be interpreted cautiously due to the limited number of eyes and individuals studied, the results presented here suggest that the explorers did not experience any detremental effects to their eyes or visual function and that future expeditions in this context are likely to be safe.

At the post-expedition follow-up examination, we identified very little change in VA, IOP, colour and contrast sensitivity, and identified no visual field defects or worsening of refractive error. Furthermore, anterior and posterior segment examination was normal. Our results show that an 8-month period spent in Antarctica is unlikely to affect these ocular parameters in those with healthy eyes. It is possible that a more prolonged stay in Antarctica, repeated visits to the polar regions or visits by those with a significant ophthalmic history may lead to ocular damage. An unlikely but possible scenario is that the eyes had time to return to normal between the end of the Antarctic expedition and our post-expedition follow-up. However, this is unlikely as we would expect to find intra-expedition changes to these visual parameters. It is also possible for further damage to reveal itself in the months or years after the post-expedition follow-up. Future studies should aim to follow-up Antarctic explorers years after the Antarctic expeditions to identify if any new pathology has developed.

Studies in mountain climbers have found hyperopic shifts at very high altitude, particularly in those with refractive surgery [4]. The first visual acuity measurement of explorer D’s right eye taken in May during the expedition showed a change of 0.31 LogMAR units, compared to his pre-expedition test. This explorer had previous refractive surgery on this eye. Whilst it is possible that this decrease in visual acuity was due to a transient change in the refractive error of the explorer’s right eye, we felt this would be unlikely, as no coresponding change was found on autorefraction and is more likely to be a measurement error.

Many studies have demonstrated a slight increase in IOP at high altitude (2500 m) and a subsequent decrease at extremely high altitude (5000 m), suggesting that the reduction in the partial pressure of oxygen at very high altitude leads to an impairment of aqueous humour production [1, 16]. One study also investigated the effects of low temperatures on IOP. Gerald et al. demonstrated that blowing cold air at a temperature of − 19°C at one eye caused a significant reduction in IOP compared to the fellow eye, which acted as a control. They hypothesised that this reduction in IOP may be due to a decrease in episcleral venous pressure through local arteriolar constriction [17]. However, we found no clinically relevent changes in IOP during the expedition or at the post-expedition assesment. Whilst it is possible there was a change in the IOPs in our explorers, their IOPs may have had time to return to normal by the time our measurements were taken indoors. Similarly, we found no haemorragic retinopathy that can develop in climbers who reach very high altitude [5–7].This is most likely due to the explorers in this expedition reaching an altitude of 2752 m compared to altitudes of over 4000 m reached in other studies [1].

The OCT-measured retinal thickness can vary in the healthy human eye by up to 10% and is not thought to represent a true underlying change [18]. We found a statistically significant decrease in retinal thickness in all retinal sectors in all eyes (other than the macula). Whilst the decrease was well under the normal variation of 10% the same OCT measuring device was used for the pre and post-expedition measurements. Furthrmore the decrease was across every eye in all explorers. However, we found no corresponding change in visual acuity and no visual field defects were identified; therefore it is very unlikely that the retinal thinning had a detrimental effect on visual function. Further studies should investigate whether repeated visits to the polar regions have a larger effect on retinal thickness and should aim to follow up scientists who spend longer periods of time in the polar regions.

## Conclusions

In this study, 10 eyes were thoroughly investigated for changes in ocular parameters during an 8 month expedition on the Antarctic plateau. No clinically relevant pathological changes were identified and these results suggest that similar future expeditions and overwintering in Antarctica and the polar regions can be conducted without significant risk of ocular damage.

## Abbreviations

IOP: Intraocular pressure
OCT: Optical coherence tomography
TAWT: Trans-Antarctic winter traverse
UV: Ultraviolet
VA: Visual acuity
